# Quasielastic neutron scattering of brucite to analyse hydrogen transport on the atomic scale

**DOI:** 10.1107/S1600576718013158

**Published:** 2018-10-25

**Authors:** Takuo Okuchi, Naotaka Tomioka, Narangoo Purevjav, Kaoru Shibata

**Affiliations:** a Institute for Planetary Materials, Okayama University, Misasa, Tottori 682-0193, Japan; b Kochi Institute for Core Sample Research, Japan Agency for Marine-Earth Science and Technology (JAMSTEC), Nankoku, Kochi 783-8502, Japan; c Hiroshima Institute of Plate Convergence Region Research, Hiroshima University, Hiroshima 739-8526, Japan; d Materials and Life Science Division, J-PARC Center, Japan Atomic Energy Agency, Tokai, Ibaraki 319-1195, Japan

**Keywords:** hydrogen transport, quasielastic neutron scattering, QENS, hydrous minerals, brucite, transmission electron microscopy

## Abstract

It is demonstrated that quasielastic neutron scattering is a novel and effective method to analyse atomic scale hydrogen transport processes occurring within a mineral crystal lattice. The method was previously characterized as sensitive for analysing the transport frequency and distance of highly diffusive hydrogen atoms or water molecules in condensed matter. Here are shown the results of its application to analyse the transport of much slower hydrogen atoms which are bonded into a crystal lattice as hydroxyls. Two types of hydrogen transport process were observed in brucite, Mg(OH)_2_: a jump within a single two-dimensional layer of the hydrogen lattice and a jump into the next nearest layer of it. These transport processes observed within the prototypical structure of brucite have direct implications for hydrogen transport phenomena occurring within various types of oxides and minerals having layered structures.

## Abstract 2

Quasielastic neutron scattering is applied to analyse the atomic scale hydrogen transport processes occurring within the crystal lattices of hydrous minerals. Two types of transport processes were observed in Mg(OH)_2_, which has a prototypical layered hydrogen lattice structure.

## 1. Introduction   

**Figure 1:**
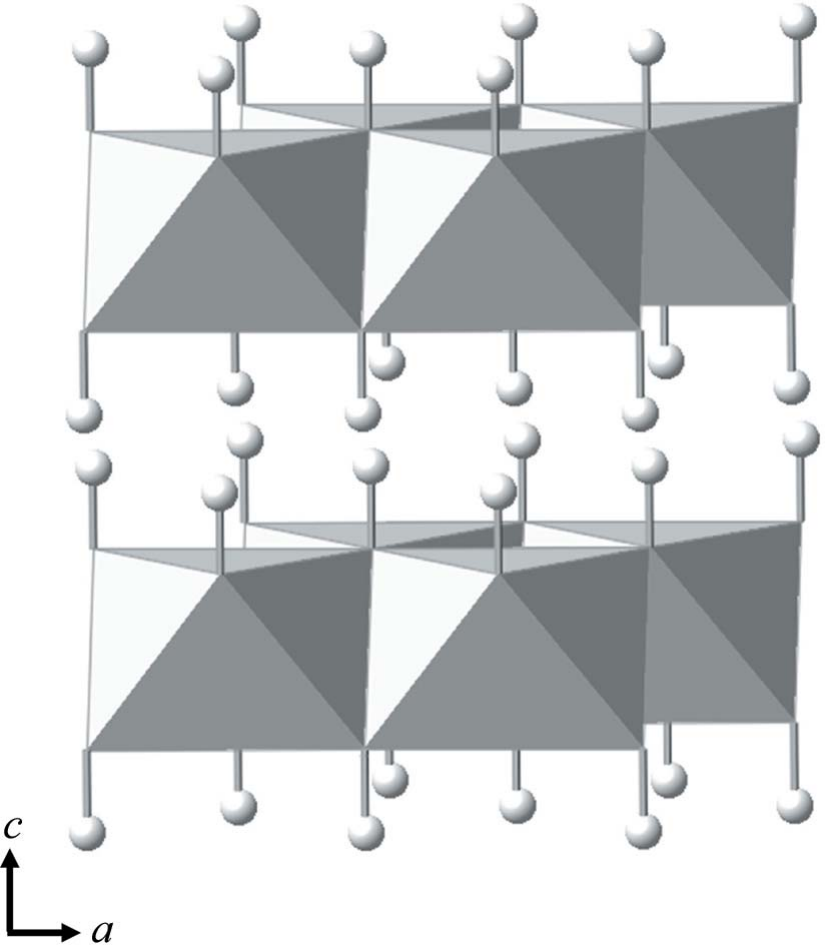
The crystal structure of brucite, Mg(OH)_2_, which belongs to space group 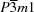. Layers of edge-sharing MgO_6_ octahedra are stacked along the *c* axis. White circles show hydrogen atoms forming two-dimensional layers on both sides of the layers of MgO_6_ octahedra.

Atomic scale hydrogen transport processes occurring within the crystal lattices of oxides and minerals are important factors controlling the kinetic behaviour of their hydration and dehydration reactions, and affecting the physical properties of the reaction products. Brucite, Mg(OH)_2_, is a hydroxide having the prototypical layered structure and so is ideal for studying the consequences of such hydrogen transport phenomena (Fig. 1 ▸). The structure has been proved to be stable over a broad range of pressure and temperature (Kruger *et al.*, 1989 ▸; Parise *et al.*, 1994 ▸; Partin *et al.*, 1994 ▸; Desgranges *et al.*, 1996 ▸; Horita *et al.*, 2010 ▸; Xu *et al.*, 2013 ▸; Okuchi *et al.*, 2014 ▸), and hydrogen transport parameters have been extensively studied in previous work (Freund & Hösen, 1977 ▸; Freund & Wengeler, 1980 ▸; Gasc *et al.*, 2011 ▸; Guo *et al.*, 2013 ▸). The consequences of the hydrogen transport processes in brucite have also been extensively studied using electron microscopy, and significant evidence of hydrogen transport has been observed within the crystal lattice, such as lamellar dehydroxylation and rehydroxylation textures (Anderson & Horlock, 1962 ▸; McKelvy *et al.*, 2001 ▸; Gomez-Villalba *et al.*, 2016 ▸; Pimminger *et al.*, 2016 ▸). Controlling such textures is essential for the industrial applications of the oxide produced after calcination of brucite (Shand, 2006 ▸; Gomez-Villalba *et al.*, 2017 ▸). Therefore, the hydrogen transport processes in brucite are considered an important subject for in-depth analysis.

Nuclear magnetic resonance (NMR) spectroscopy and electrical conductivity (EC) measurements are known to be effective for analysing the transport of hydrogen *in situ*. A recent NMR study reported that a fraction of the hydrogen in brucite was diffusible at temperatures higher than 260 K; the molar fraction of such diffusible hydrogen gradually increased with increasing temperature and reached about one-hundredth of the total hydrogen atoms at 355 K, where the NMR measurements were terminated (Itoh & Isobe, 2016 ▸). Furthermore, previous EC results on brucite collectively indicated that it transforms into an ionic conductor at higher temperatures than ∼440 K (Freund & Hösen, 1977 ▸; Freund & Wengeler, 1980 ▸). Such diffusible hydrogen species were named ‘extrinsic protons’ or ‘defect protons’, but their transport process has not yet been clarified. The poor characterization of transport processes is due to a lack of information on the local structure where these protons are transferred. Here, we analyse such local transport processes using quasielastic neutron scattering (QENS), which is another distinguished method known to be potentially sensitive to the transport frequency and transport geometry of hydrogen. To the best of our knowledge, this method has not yet been applied to chemically bonded hydrogen as hydroxyls in a crystal lattice.

## 2. Experimental strategy and procedures   

### 2.1. QENS analysis of slow hydrogen transport   

Analysis of diffusive hydrogen in condensed matter has been a principal research target of the QENS method (Springer, 1972 ▸; Bee, 1988 ▸). A typical example is its application to solid-state proton conductors (Malavasi *et al.*, 2010 ▸; Karlsson, 2015 ▸). The method has also been effectively applied to analyse the motion of molecular water adsorbed into clays and zeolites, which is known to actively exchange with bulk water and is therefore highly mobile (Swenson *et al.*, 2000 ▸; Malikova *et al.*, 2006 ▸; Martins *et al.*, 2014 ▸). Another application was recently reported for water dissolving within high-temperature magmas (Yang *et al.*, 2017 ▸). Notably, the large energy transfer of incident neutrons by these diffusive hydrogen species makes the analysis rather straightforward. In contrast, the transport of covalently bonded hydrogen as hydroxyls within the crystal lattices of minerals has never been analysed by QENS, which is primarily because the energy transfer and scattering intensity have been considered too small to be a subject for such analysis. Recently, a high-resolution inelastic spectrometer was established with a drastically improved sensitivity, where a novel optical design for increasing the signal-to-background ratio was effectively coupled with a strong pulsed neutron source (Shibata *et al.*, 2015 ▸). We consider that such an advanced spectrometer could be effective to analyse the slow transport of hydroxyl hydrogens within the crystal lattice of Mg(OH)_2_.

### 2.2. QENS experimental procedures   

A commercial reagent of pure brucite powder with 0.07 µm nominal grain size [Wako Chemicals 139-13951, ≥99.9% in weight as Mg(OH)_2_] was selected to be analysed. For QENS measurements at temperatures from 180 to 330 K, 0.41 g of the reagent sample was collected and dried at 383 K in air for 1 h to remove surface-adsorbed molecular water. For QENS measurements at 430 K, 0.49 g of the reagent sample was collected and dried in a vacuum at 500 K for 1 h. These samples were separately wrapped in commercial aluminium foil (∼10 µm thick) to maintain a reagent thin-walled annular geometry in bulk. Such a geometry is effective in minimizing multiple scattering of neutrons during QENS measurements. Each foil-wrapped sample was placed in an air-tight cylinder container made of pure aluminium with a thickness of 0.25 mm and an inner diameter of 14.0 mm. Then, each cylinder container was mechanically sealed using a pure aluminium cap with a stainless steel O-ring and eight stainless steel cap screws, which was hand-fixed within a glove bag filled with dry helium gas (Fukushima *et al.*, 2018 ▸). The sealed container was then installed in a top-loading-type cryo-furnace to maintain sample temperature control during measurements.

The QENS measurements were conducted using the near-backscattering spectrometer DNA, which is installed at the Japan Proton Accelerator Research Complex (J-PARC), Materials and Life Science Experimental Facility (MLF) (Shibata *et al.*, 2015 ▸). The incident pulsed neutron beam was shaped at the chopper, scattered at the sample, filtered at the Si(111) analyser array to reach a final energy *E* = 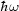 = 2084 µeV, and detected. The counter-rotating pulse-shaping choppers were set with 30 mm slit width at the open position, which provided an energy resolution of 3.6 µeV full width at half-maximum (FWHM), and an energy scan window range from −30 to 100 µeV. The total scattering function, *S*(*Q*, ω), was determined by measuring the two described samples at 180–330 and 430 K, respectively. The total resolution function, *R*(*Q*, ω), of each sample was also determined at 50 K, where we assumed that all hydrogen dynamics were frozen. The *R*(*Q*, ω) function was used for deconvolution of the instrumental function. A typical measurement duration to obtain these *S*(*Q*, ω) and *R*(*Q*, ω) functions was half a day at 300 kW proton beam power. Another cylinder container with an empty foil without a sample was measured at 300 K to subtract the background from the container. All neutron scattering event data were reduced using the *Utsusemi* software (Inamura *et al.*, 2013 ▸). The analysed *Q* [momentum transfer; *Q* = (4π/λ)sin(θ/2), where θ is the scattering angle and λ is the wavelength of the incident radiation] range was from 0.10 to 1.90 Å^−1^. A small positive Bragg reflection anomaly was induced by the large *d* spacing of brucite (*d*_001_ = 4.77 Å at 300 K), which appeared around *Q* = 1.32 Å^−1^; therefore, the range 1.30 ≤ *Q* ≤ 1.35 Å^−1^ was excluded from the analysis. The resolution of the momentum transfer was Δ*Q* ≃ 0.04 Å^−1^ around *Q* = 1.32 Å^−1^, so that the remaining *Q* range was little affected by the Bragg reflection. Except for hydrogen, all nuclei in the sample (Mg and O) induced negligible inelastic scattering intensity because of their much smaller incoherent neutron scattering lengths. Therefore, the reduced scattering function after subtracting the container background consisted solely of incoherent scattering of hydrogen in the brucite crystal structure, which involves scattering of both immobile and mobile fractions of hydrogen.

### 2.3. Transmission electron microscopy analysis   

To confirm the crystallographic properties and grain-size distribution of the described samples, the reagent was analysed by transmission electron microscopy (TEM). A small fraction of the reagent powder was heated at 383 or 523 K for 20 h in a vacuum and subsequently observed by TEM. Each powder sample was dispersed in ethanol, and the supernatants of the slurries were added dropwise onto Cu grids, each covered with a holey carbon film (Quantifoil). Each sample was then examined using a JEOL JEM-ARM-200F transmission electron microscope operated at an accelerating voltage of 200 kV at the Kochi Institute for Core Sample Research of the Japan Agency for Marine-Earth Science and Technology.

## 3. Results   

### 3.1. QENS results and analysis   

**Figure 2:**
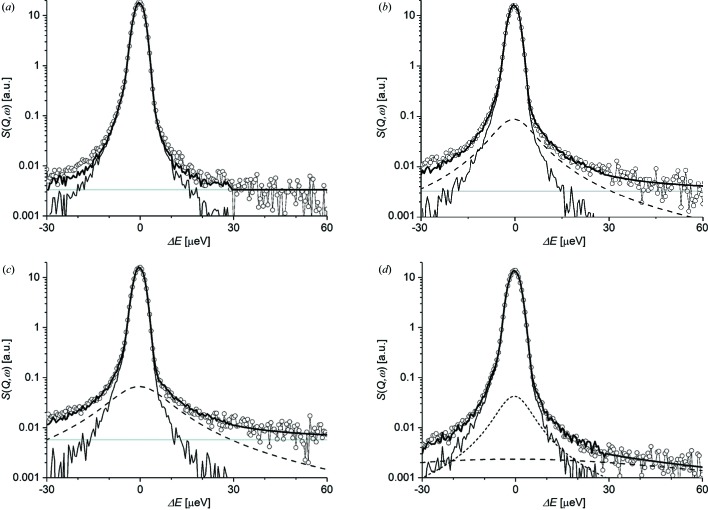
Representative *Q*-sliced scattering functions *S*(*Q*_fix_, ω) obtained at different temperatures. The *Q*_fix_ and temperature details are as follows: (*a*) 1.00 ± 0.30 Å^−1^ and 180 K, (*b*) 1.10 ± 0.15 Å^−1^ and 230 K, (*c*) *Q* = 1.15 ± 0.10 Å^−1^ and 280 K, and (*d*) *Q* = 1.625 ± 0.275 Å^−1^ and 430 K. An energy scan window range between −30 and 60 µeV is selected for each case. Open circles represent the observed functions and lines represent fitting functions for the observed ones; thin black lines are the resolution function *R*, long- and short-dashed lines are the Lorentz functions *L*(Γ_1_, ω) and *L*(Γ_2_, ω), respectively, thick grey lines are the background BG, and thick black solid lines are the sum of all these functions. The resolution function was convoluted in the energy scan window range between −30 and 30 µeV.

Fig. 2 ▸ shows representative scattering functions with some limited *Q* ranges. Except for the case at 180 K, the narrow elastic component of static hydrogen and the broad quasielastic component of mobile hydrogen were detected simultaneously. The former component consisted of a delta function, δ(ω), while the latter component consisted of one or more Lorentz functions, *L*[Γ_*n*_(*Q*), ω], where Γ_*n*_ is the half-width at half-maximum, HWHM, of the *n*th function, and ω = 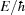. Note that upon detection these functions were convoluted with the relevant resolution function involving the instrumental effects. As indicated by the previous NMR measurements for brucite, the broad quasielastic component had a much smaller scattering intensity than the narrow elastic component, so that the enhanced spectrometer sensitivity was essential to detect the former component.

To induce such a QENS effect within a well ordered crystalline lattice, hydrogen is chemically bonded to its crystallographic site and also jumps to another site through one or more transport processes on the atomic scale. Then, the scattering function at a near-fixed *Q* (*Q*_fix_) is described as follows (Bee, 1988 ▸; Springer, 1972 ▸; Shibata *et al.*, 2015 ▸; Seto *et al.*, 2017 ▸): 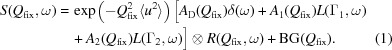
The 〈*u* ^2^〉 term is the mean-square displacement (or atomic displacement parameter), and *A*_D_, *A*_1_ and *A*_2_ are the area intensities of the delta function and first and second Lorentz functions, respectively. Owing to signal-to-noise ratio limitations, we constrain the number of Lorentz functions to be one or two. BG is a flat background mainly coming from phonon scattering. Some of these parameters change significantly with *Q*; therefore, to apply this relationship, *S*(*Q*, ω) and *R*(*Q*, ω) were sectioned into a series of functions that cover the limited *Q* ranges.

**Figure 3:**
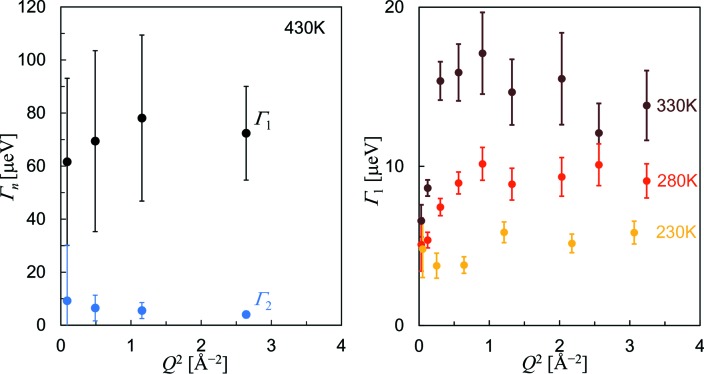
The line widths (HWHM) for the Lorentz functions *L*(Γ_1_, ω) and *L*(Γ_2_, ω) plotted as a function of *Q* ^2^. These widths commonly show asymptotic behaviour at larger *Q*, so that equation (3) is satisfied.

Figs. 3 ▸ and 4 ▸ show Γ_*n*_ and *A_n_* at different temperatures. They were optimized separately for each of the sectioned scattering functions with near-fixed *Q*, and then plotted altogether. The *QENSfit* software provided for the DNA spectrometer was used for these fits. At 180 K, the scattering function did not contain any resolvable Lorenz component (*A*_1_ = *A*_2_ = 0; Fig. 2 ▸ *a*). At temperatures from 230 to 330 K, the scattering function contained one Lorenz component (*A*_1_ > 0, *A*_2_ = 0; Figs. 2 ▸ *b* and 2 ▸ *c*). At 430 K, the function contained two Lorentz components (*A*_1_ > 0, *A*_2_ > 0; Fig. 2 ▸ *d*).

**Figure 4:**
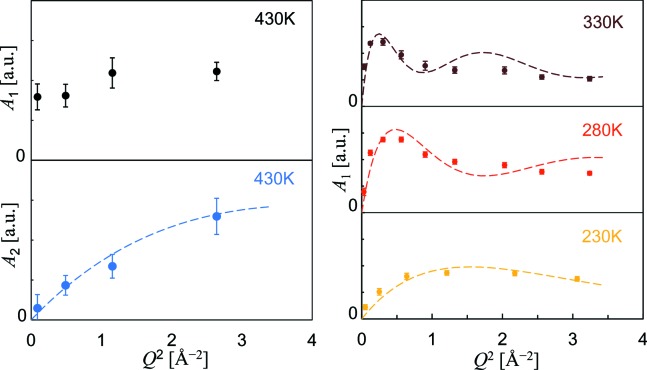
The area intensities for the Lorentz functions *A*_1_ and *A*_2_ plotted as a function of *Q* ^2^. The broken lines show the fit results using equation (2). For the fitting of *A*_1_ at temperatures from 280 to 330 K, the atomic displacement parameters of hydrogen along the transport direction (almost perpendicular to the OH bond) are set to be consistent with the neutron powder diffraction result (0.04 Å^2^ at 230 K, 0.05 Å^2^ at 280 K and 0.06 Å^2^ at 330 K; Chakoumakos *et al.*, 2013 ▸). The *A*_2_ profile at 430 K is reproducible only when *d*_2_ is much smaller than 3.14 Å, indicating that the process inducing this profile is a transport into the next layer. Therefore, the fitting of *A*_2_ at 430 K was made with a zero atomic displacement parameter for hydrogen, because its vibration along the transport direction (almost along the OH bond) is much more restricted.

For further analysis, we focus on the observed oscillating feature of *A*_1_ as a function of *Q* (Fig. 4 ▸), which is evidence of a localized transport process between a few distinct hydrogen sites, such as hydrogen’s motion during molecular reorientations [ch. 6 in the book by Bee (1988 ▸)]. Therefore, we adopted the simplest two-site jump model for the transport process, where hydrogen atoms move back and forth between two equivalent sites separated at a root-mean-square dis­placement *d*_*n*_ (*n* = 1 or 2). Considering the Γ_1_ profiles as observed in Fig. 3 ▸, this model is indeed one of the most preferred, because the other jump models involving four or more sites inevitably induce large oscillations in the corresponding Γ profiles; the two- (or three-)site model certainly reproduces the relatively flat Γ profiles. We note that the analysis of *d*_*n*_ is not affected very much by the selection of two or three sites, and also that the analysis of τ_*n*_ is independent of the selection of site numbers. Thus, it is reasonable to represent the hydrogen transport process by the simplest two-site model.

The hydrogen at the filled site oscillates around its equilibrium position for τ_*n*_ (*n* = 1 or 2) and then jumps over the distance *d*_*n*_ into the empty site. The resulting equation is
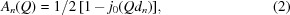
where *j*_0_ is a Bessel function of zero order (Bee, 1988 ▸). To find *d*_*n*_ at each temperature, the model *A_n_* functions given in equa­tion (2) were fitted to the observed values by adjusting *d*_*n*_ (Fig. 4 ▸).

**Figure 5:**
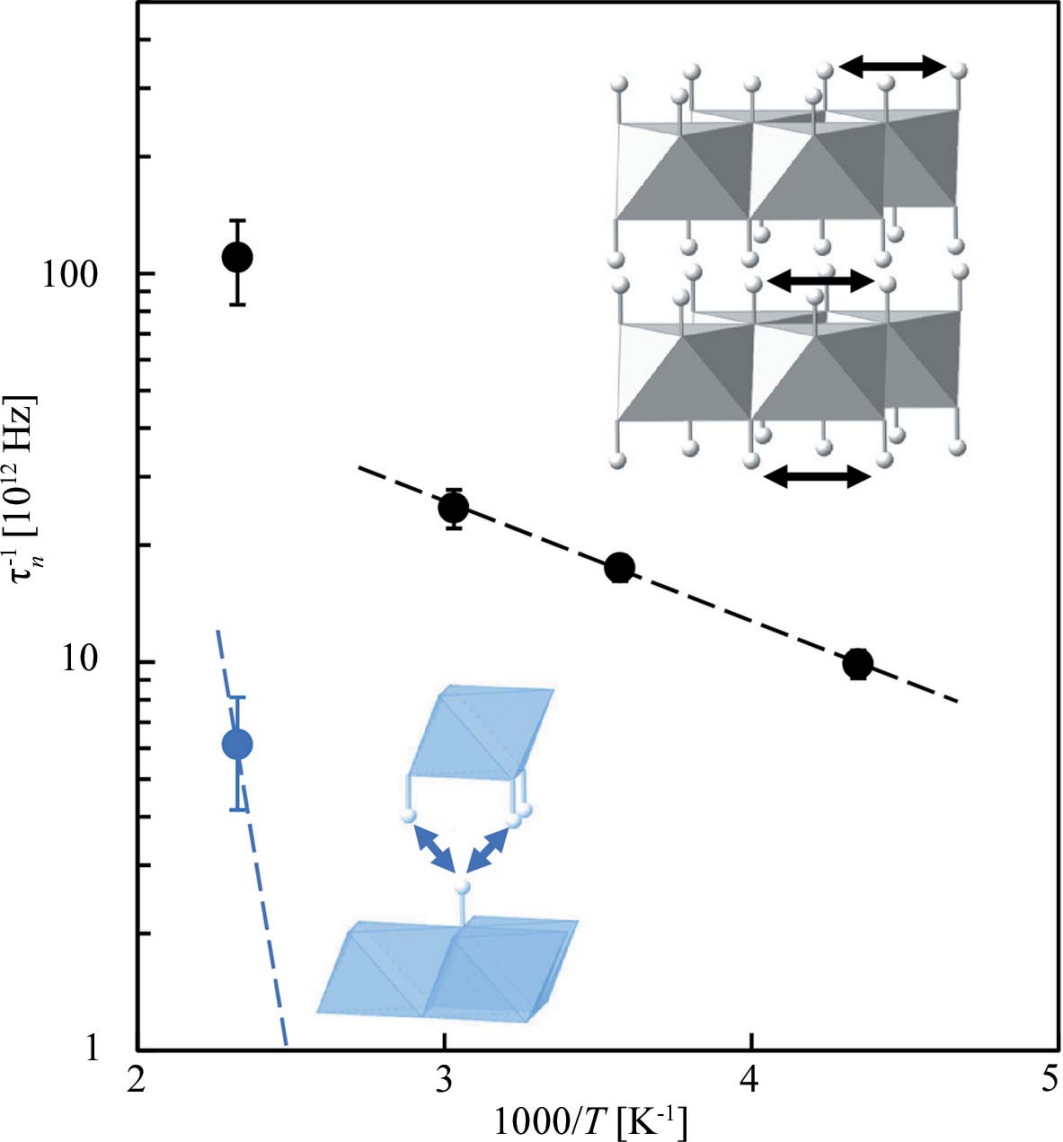
Jump frequencies τ_*n*_^−1^ of two hydrogen transport processes as a function of reciprocal temperature. To increase the statistics and reduce the uncertainty in Γ_*n*_, the observed *S*(*Q*, ω) at all temperatures were resectioned and reanalysed over a wide *Q* range from 1.35 to 1.90 Å^−1^. The black dashed line shows the slope of τ_1_^−1^ at temperatures between 230 and 330 K, which gives an activation energy of 0.061 eV. The blue dashed line shows the expected slope of τ_2_^−1^ having an activation energy of 1.0 eV as determined by EC measurements. The insets show the hydrogen transport pathways (black arrows for the τ_1_^−1^ process and blue arrows for the τ_2_^−1^ process).

By taking into account that the root-mean-square displace­ment of the oscillation, 〈*u* ^2^〉^1/2^, is much smaller than that of the jump, another equation is deduced,
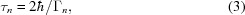
which is effective for *Qd* > 1 (Springer, 1972 ▸). The value of τ_*n*_^−1^ was calculated using this equation and plotted as a function of reciprocal temperature (Fig. 5 ▸), where the activation energy of hydrogen transport was evaluated. Table 1 ▸ summarizes τ_*n*_, *d*_*n*_ and the fractional intensity of *A*_*n*_ compared with the total intensity *A*_total_ = *A*_D_ + *A*_1_ + *A*_2_.

### 3.2. TEM results   

**Figure 6:**
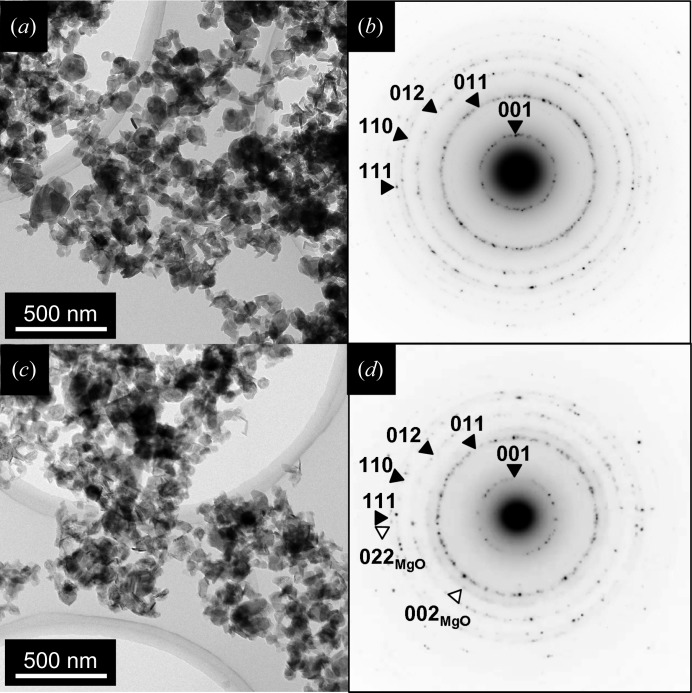
Transmission electron micrographs and selected-area electron diffraction patterns from aggregates of brucite grains. The samples were heated at (*a*), (*b*) 383 K or (*c*), (*d*) 523 K. Each sample consists of brucite grains smaller than 200 nm in size. Diffraction rings indicated with filled triangles are from brucite. The 523 K sample also shows very weak diffraction rings from periclase, MgO (indicated with open triangles). The periclase grains are artefacts resulting from dehydration of the brucite due to electron-beam damage during TEM observation.

Fig. 6 ▸ shows transmission electron micrographs and corresponding selected-area electron diffraction (SAED) patterns of the brucite powder samples. The grain sizes were measured for 50 grains in each fraction heated at 383 and 523 K. They ranged from 40 to 200 nm and were indistinguishable between these fractions. From the SAED patterns, all sample grains had a confirmed good crystalline brucite structure, so that the hydrogen transport processes analysed by QENS occurred within the crystalline lattice. By referring to these TEM results, we excluded the possibility of irreversible degradation of the brucite structure with increasing temperature.

## 4. Discussion   

It is confirmed that a fraction of hydrogen in the brucite lattice is diffusible even at 230 K, although this is ∼400 K lower than the dehydration temperature (Garn *et al.*, 1978 ▸; Shand, 2006 ▸). The occurrence of such diffusible hydrogen in brucite in this temperature regime had been repeatedly proposed on the basis of NMR studies, but evidence for details of the transport process was scarce (Saito & Kotera, 1963 ▸; Sears *et al.*, 1988 ▸; Itoh & Isobe, 2016 ▸). Because the reported activation energy of hydrogen transport (0.082 eV; Itoh & Isobe, 2016 ▸) was almost comparable to that determined by QENS in the current study (0.061 eV; Fig. 5 ▸), we conclude that both NMR and QENS methods are sensitive to the same transport process, which is proved to be active down to at least 230 K. The process observed by NMR had been thought to be facilitated by some lattice defects, because its activation energy was very much smaller than that of proton-induced electronic conduction through the regular crystalline lattice of Mg(OH)_2_ (Freund & Wengeler, 1980 ▸; Itoh & Isobe, 2016 ▸). In this latest work, we have now obtained the jump distance *d*_1_ = 2.9 Å of the defect-related transport process at 230 K. This distance indicates that the transport first occurs between the two closest hydrogen sites within a single OH^−^ layer of the brucite lattice (3.14 Å at 200 K). There is no other geometry for hydrogen to jump across such a distance. In addition, the observed *d*_1_ at 280 and 330 K increases with increasing temperature, reaching the second-closest (*d* = 5.4 Å), third-closest (*d* = 6.3 Å) and even more distant sites within the same OH^−^ layer of brucite. As the jump distance increases with increasing temperature from 230 to 330 K, so the jump frequency τ_1_ also increases smoothly and continuously, from which we have reconfirmed that the transport process for *d*_1_ and τ_1_ is definable by a single activation-energy process. We have two possibilities to describe this lattice-defect-facilitated transport process, which is either excess-proton transport or proton-defect transport. While the protons in the former process feel a proton–proton repulsive interaction during transport, the protons in the latter process rather feel a proton–defect attractive interaction. Therefore, with the former process, it is much easier for the protons to jump longer distances. The process in brucite is most probably the excess-proton type, as this was previously observed to induce proton conductivity in another layered hydrous mineral, kaolinite (Maiti & Freund, 1981 ▸).

The activation energy of electrical conductivity in dry brucite is around 1.0 eV (Freund & Hösen, 1977 ▸; Freund & Wengeler, 1980 ▸; Gasc *et al.*, 2011 ▸), which is far larger than that of the transport process responsible for τ_1_ and *d*_1_. We also note that such conductivity in brucite has been observed only at temperatures beyond 440 K. To address this issue in more depth, the QENS result at the comparable temperature (430 K) is particularly valuable; it suggests the emergence of another transport process, with a relaxation time τ_2_ and a jump distance *d*_2_ that are significantly different from τ_1_ and *d*_1_. In particular, *d*_2_ cannot be comparable to or larger than 3.14 Å (Fig. 5 ▸), which indicates that hydrogen transport through this second process occurs from one hydroxyl (OH^−^) layer to the other (opposing) OH^−^ layer. This conclusion is based on the distance of 1.93 Å between the two closest interlayer sites, which is the only candidate with such a short jump distance; the fitting curve of *A*_2_ with *d*_2_ = 1.93 Å shows quantitative consistency with this conclusion. While such a process has never been scanned by NMR, it was possible to detect it in the EC measurements, where it was considered to be the motion of a negatively charged point defect of the proton (H′) (Freund & Wengeler, 1980 ▸). Such a proton defect is formed from an OH^−^, together with the production of an excess proton. The excess proton may quickly diffuse out, while the remaining proton defect slowly moves step by step by receiving another proton from its closest OH^−^ that belongs to the other (opposing) layer. Because of the large activation energy required to remove the proton from OH^−^, this process would not be observed by QENS at temperatures lower than 430 K (Fig. 5 ▸). We also note that the observed Γ_1_ at 430 K was almost as large as the energy scan window range of the spectrometer, so that τ_1_^−1^ at this temperature was relatively uncertain; however, this observation does not preclude τ_1_^−1^ non-continuously increasing from temperatures lower than 330 K. The activation of defect motion would produce this result because that motion simultaneously rearranges the OH^−^ dipole orientation distributions of the two opposing OH^−^ layers, *i.e.* the layers the defect moves from and to, by changing the electrostatic interaction between the point charge and surrounding dipoles. This rearrangement may further stimulate the relevant transport process for τ_1_^−1^ within these two layers.

## 5. Conclusions   

It has been demonstrated that QENS analysis of hydrogen in the crystal structures of minerals is uniquely effective in elucidating the frequency and geometry of its transport processes. Because of the prototypical structure of brucite consisting of layered hydrogen lattices, its analysis using QENS provides information that may be applicable to hydrogen transport processes occurring in a variety of oxides and minerals having similar structures. For example, the hydrogen-site geometry in a high-density mineral existing in the Earth’s deep mantle (dense hydrous magnesium silicate phase E, nominal composition ∼ Mg_2_SiO_2_(OH)_4_, space group 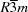; Tomioka *et al.*, 2016 ▸) is very close to that of brucite compressed along its *c* axis (Parise *et al.*, 1994 ▸; Okuchi *et al.*, 2014 ▸). By isotope diffusion analysis, it has been proved that the rate of hydrogen transport in brucite increases with compression (Guo *et al.*, 2013 ▸), so it is expected that hydrogen transport in this deep-Earth mineral can also be accelerated to be detectable by QENS. We are working on systematic syntheses and subsequent QENS analyses of such deep-Earth minerals (Okuchi *et al.*, 2015 ▸), which have pre­established hydrogen-site geometries from neutron diffraction studies (Sano-Furukawa *et al.*, 2011 ▸; Purevjav *et al.*, 2014 ▸, 2016 ▸, 2018 ▸; Tomioka *et al.*, 2016 ▸).

## Figures and Tables

**Table 1:** Summary of parameters of hydrogen transport processes in brucite

Temperature (K)	τ_1_ (ps)	*d*_1_ (Å)	*A*_1_/*A*_total_	τ_2_ (ps)	*d*_2_ (Å)	*A*_2_/*A*_total_
230	100 (10)	2.9	0.03			
280	57 (5)	5.4	0.04			
330	40 (5)	7.8	0.04			
430	9 (3)	>10	0.01	160 (80)	∼1.9	0.01

## References

[bb1] Anderson, P. J. & Horlock, R. F. (1962). *Trans. Faraday Soc.* **58**, 1993–2004.

[bb2] Bee, M. (1988). *Quasielastic Neutron Scattering, Principles and Applications in Solid State Chemistry, Biology and Materials Science.* Bristol, Philaelphia: Adam Hilger.

[bb3] Chakoumakos, B. C., Horita, J. & Garlea, V. O. (2013). *Am. Mineral.* **98**, 1–6.

[bb4] Desgranges, L., Calvarin, G. & Chevrier, G. (1996). *Acta Cryst.* B**52**, 82–86.

[bb5] Freund, F. & Hösen, R. (1977). *Ber. Bunsenges. Phys. Chem.* **81**, 39–43.

[bb6] Freund, F. & Wengeler, H. (1980). *Ber. Bunsenges. Phys. Chem.* **84**, 866–873.

[bb7] Fukushima, Y., Yamada, T., Tamura, K. & Shibata, K. (2018). *Appl. Clay Sci.* **155**, 15–19.

[bb8] Garn, P. D., Kawalec, B. & Chang, J. C. (1978). *Thermochim. Acta*, **26**, 375–381.

[bb9] Gasc, J., Brunet, F., Bagdassarov, N. & Morales-Flórez, V. (2011). *Phys. Chem. Miner.* **38**, 543–556.

[bb10] Gomez-Villalba, L. S., Sierra-Fernandez, A., Milosevic, O., Fort, R. & Rabanal, M. E. (2017). *Adv. Powder Technol.* **28**, 61–72.

[bb11] Gomez-Villalba, L. S., Sierra-Fernandez, A., Rabanal, M. E. & Fort, R. (2016). *Ceram. Int.* **42**, 9455–9466.

[bb12] Guo, X. Z., Yoshino, T., Okuchi, T. & Tomioka, N. (2013). *Am. Mineral.* **98**, 1919–1929.

[bb13] Horita, J., dos Santos, A., Tulk, C., Chakoumakos, B. & Polyakov, V. (2010). *Phys. Chem. Miner.* **37**, 741–749.

[bb14] Inamura, Y., Nakatani, T., Suzuki, J. & Otomo, T. (2013). *J. Phys. Soc. Jpn*, **82**, SA031.

[bb15] Itoh, Y. & Isobe, M. (2016). *J. Phys. Soc. Jpn*, **85**, 034602.

[bb16] Karlsson, M. (2015). *Phys. Chem. Chem. Phys.* **17**, 26–38. 10.1039/c4cp04112g 25354017

[bb17] Kruger, M. B., Williams, Q. & Jeanloz, R. (1989). *J. Chem. Phys.* **91**, 5910–5915.

[bb18] Maiti, G. C. & Freund, F. (1981). *Clay Miner.* **16**, 395–413.

[bb19] Malavasi, L., Fisher, C. A. J. & Islam, M. S. (2010). *Chem. Soc. Rev.* **39**, 4370–4387. 10.1039/b915141a 20848015

[bb20] Malikova, N., Cadène, A., Marry, V., Dubois, E. & Turq, P. (2006). *J. Phys. Chem. B*, **110**, 3206–3214. 10.1021/jp056954z 16494330

[bb21] Martins, M. L., Gates, W. P., Michot, L., Ferrage, E., Marry, V. & Bordallo, H. N. (2014). *Appl. Clay Sci.* **96**, 22–35.

[bb22] McKelvy, M. J., Sharma, R., Chizmeshya, A. V. G., Carpenter, R. W. & Streib, K. (2001). *Chem. Mater.* **13**, 921–926.

[bb23] Okuchi, T., Purevjav, N., Tomioka, N., Lin, J. F., Kuribayashi, T., Schoneveld, L., Hwang, H., Sakamoto, N., Kawasaki, N. & Yurimoto, H. (2015). *Am. Mineral.* **100**, 1483–1492.

[bb24] Okuchi, T., Tomioka, N., Purevjav, N., Abe, J., Harjo, S. & Gong, W. (2014). *High. Pressure Res.* **34**, 273–280.

[bb25] Parise, J. B., Leinenweber, K., Weidner, D. J., Tan, K. & Von Dreele, R. B. (1994). *Am. Mineral.* **79**, 193–196.

[bb26] Partin, D. E., O’Keefe, M. & Von Dreele, R. B. (1994). *J. Appl. Cryst.* **27**, 581–584.

[bb27] Pimminger, H., Habler, G., Freiberger, N. & Abart, R. (2016). *Phys. Chem. Miner.* **43**, 59–68.

[bb28] Purevjav, N., Okuchi, T., Tomioka, N., Abe, J. & Harjo, S. (2014). *Geophys. Res. Lett.* **41**, 6718–6724.

[bb29] Purevjav, N., Okuchi, T., Tomioka, N., Wang, X. P. & Hoffmann, C. (2016). *Sci. Rep.* **6**, 34988. 10.1038/srep34988 PMC5057097 27725749

[bb30] Purevjav, N., Okuchi, T., Wang, X., Hoffmann, C. & Tomioka, N. (2018). *Acta Cryst.* B**74**, 115–120.

[bb31] Saito, T. & Kotera, Y. (1963). *Bull. Chem. Soc. Jpn*, **36**, 474–475.

[bb32] Sano-Furukawa, A., Kuribayashi, T., Komatsu, K., Yagi, T. & Ohtani, E. (2011). *Phys. Earth Planet. Inter.* **189**, 56–62.

[bb33] Sears, R. E. J., Kaliaperumal, R. & Manogaran, S. (1988). *J. Chem. Phys.* **88**, 2284–2288.

[bb34] Seto, H., Itoh, S., Yokoo, T., Endo, H., Nakajima, K., Shibata, K., Kajimoto, R., Ohira-Kawamura, S., Nakamura, M., Kawakita, Y., Nakagawa, H. & Yamada, T. (2017). *Biochim. Biophys. Acta Gen. Subj.* **1861**, 3651–3660. 10.1016/j.bbagen.2016.04.025 27156489

[bb35] Shand, M. A. (2006). *The Chemistry and Technology of Magnesia.* Hoboken: John Wiley and Sons.

[bb36] Shibata, K., Takahashi, N., Kawakita, Y., Matsuura, M., Yamada, T., Tominaga, T., Kambara, W., Kobayashi, M., Inamura, Y., Nakatani, T., Nakajima, K. & Arai, M. (2015). *JPS Conf. Proc.* **8**, 036022.

[bb37] Springer, T. (1972). *Quasielastic Neutron Scattering for the Diffusive Motions in Solids and Liquids.* Heidelberg: Springer-Verlag.

[bb38] Swenson, J., Bergman, R. & Howells, W. S. (2000). *J. Chem. Phys.* **113**, 2873–2879.

[bb39] Tomioka, N., Okuchi, T., Purevjav, N., Abe, J. & Harjo, S. (2016). *Phys. Chem. Miner.* **43**, 267–275.

[bb40] Xu, H. W., Zhao, Y. S., Hickmott, D. D., Lane, N. J., Vogel, S. C., Zhang, J. Z. & Daemen, L. L. (2013). *Phys. Chem. Miner.* **40**, 799–810.

[bb41] Yang, F., Hess, K. U., Unruh, T., Mamontov, E., Dingwell, D. B. & Meyer, A. (2017). *Chem. Geol.* **461**, 152–159.

